# It Is Time to Make Policy for Healthier Food Environments in Australian Universities

**DOI:** 10.3390/nu10121909

**Published:** 2018-12-04

**Authors:** Yumeng Shi, Qing Wang, Courtney Norman, Margaret Allman-Farinelli, Stephen Colagiuri

**Affiliations:** 1 The Boden Institute of Obesity, Nutrition, Exercise & Eating Disorders, Charles Perkins Centre, The University of Sydney, Sydney, NSW 2006, Australia; stephen.colagiuri@sydney.edu.au; 2 Nutrition and Dietetics Group, School of Life and Environmental Sciences, Charles Perkins Centre, The University of Sydney, Sydney, NSW 2006, Australia; qwan5315@uni.sydney.edu.au (Q.W.); courtneyleighnorman@hotmail.com (C.N.); margaret.allman-farinelli@sydney.edu.au (M.A.-F.)

**Keywords:** food environment, food outlets, vending machines, university, college, young adults, food policy

## Abstract

The obesogenic food environment is likely driving excessive weight gain in young adults. Our study aimed to investigate the nutritional quality of current food and drink offerings in an Australian university. This cross-sectional study included baseline environmental audits of 30 food outlets and 62 vending machines across campus. A recent food and drink benchmark for health facilities by state government was used to classify the food and beverage offerings. It recommended food outlets and vending machines to offer at least 75% ‘Everyday’ (healthy) and less than 25% ‘Occasional’ (less healthy) foods and drinks. Sugary drinks and options with large portion sizes and unhealthy ingredients should be removed from sale. Only two beverage vending machines and none of the food outlets met the full recommendations. The overall proportions of Everyday and Occasional foods in food outlets were 35% and 22%, respectively with 43% falling into the category that should not be sold. Sugary drinks occupied a third of beverage varieties in outlets and 38% of beverage slots in vending machines. The current university food environment was poorly compliant with the existing benchmark. Specific food policy in the university setting may be needed to make healthier choices more accessible to young adults.

## 1. Introduction

National population-based studies in Australia have found that young adults experienced a greater increase in weight and waist circumference [1] and a greater annual increase in body mass index than other adult generations over the same follow-up period [2]. This trend is likely driven in part by continued exposure to the contemporary obesogenic food environment throughout their entire life, and evidence shows young adults have the poorest quality diets [3]. 

More than 1.5 million students aged 15–34 years and 42% of adults aged 20–24 years enrolled in Australian tertiary education institutions in 2017 [4]. However, a previous environmental audit in Australian universities indicated undesirable nutritional quality of food outlets on campuses [5]. A previous study at an Australian university found that the top priority for students was to provide healthier foods and to make them available at lower prices [6]. Students in Canada and Belgium similarly suggested more healthy offerings in the restaurants on campus, and they would tend to choose nutritious choices if readily available with a lower price [7,8]. A systematic review of food environment interventions in tertiary institutions also indicated that some approaches may have positive impacts on the food purchasing behaviours of young adults, such as pricing strategies and increasing the accessibility to healthy choices [9].

There are a variety of guidelines and benchmarks for healthier food environments in schools and workplaces implemented internationally [10,11]. Within Australia, different states have implemented a number of systems for determining healthy and unhealthy food choices for sale [12,13]. In New South Wales (NSW), the Ministry of Health has introduced ‘Healthy Food and Drink in Health Facilities for Staff and Visitors Framework’ [14]. The Framework provides a guideline to improve the offering of healthy food choices in food outlets and vending machines in health facilities. The ‘Food and Drink Benchmark’ in the Framework is stated to be underpinned by the Australian Dietary Guidelines [15]. To assess the product quality, the Health Star Rating (HSR) system is applied in the Benchmark, which is a voluntary front-of-pack labelling system to classify food and drinks according to the nutrient contents and ingredients [16].

This study aims to (i) conduct a baseline audit of vending machines and food outlets in the university to check the compliance of available foods and drinks with the Food and Drink Benchmark, and to (ii) provide recommendations for improvements to close the gap between the current university food environment and the one that would enable healthier food consumption.

## 2. Materials and Methods 

### 2.1. Design

This cross-sectional study involved a food environment mapping of food outlets and vending machines across a large urban university in Australia. The food outlet audit was conducted between January and March 2018, and vending machines were audited between August and September 2017. This study did not involve human subjects and was defined as negligible risk according to the National Statement on Ethical Conduct in Human Research [17], and thus no ethics approval was required by the institutional Human Research Ethics Committee.

### 2.2. Classification Criteria

The ‘Ready Reckoner’ in the Benchmark classifies items in each food and drink category into two groups: ‘Everyday’ that constitutes the healthier options and ‘Occasional’ that are less healthy options (see Table 1). However, if the criteria that concern the portion size, HSR and basic ingredients could not be met within the groups, the items are classified as ‘Everyday does not meet criteria’ and ‘Occasional does not meet criteria’. Sugary drinks are classified as an additional subgroup.

Everyday foods and drinks are meals, snacks, and drinks made from foods in the five main food groups that are vegetables and legumes; fruit; milk, yoghurt, cheese and alternatives; lean meat, poultry, fish, eggs, tofu, nuts and seeds; grain foods including bread, pasta, and rice. Occasional choices are mostly foods high in saturated fat, added sugars, and/or salt and often have little nutritional value [14]. The Benchmark for NSW health facilities requires an HSR of 3.5 stars or above for packaged foods and drinks including muesli and snack bars; lightly salted or flavoured popcorn, nuts, seeds and legume snacks; savoury biscuits; salty snacks; breakfast cereals; packaged ready-to-eat meals; instant flavoured noodles; flavoured milk and liquid breakfast drinks [14]. Specific portion limits were recommended for some Everyday and all Occasional choices (listed in Table 1). The ingredients that are listed as ‘do not use’ in the ‘Basic Ingredients List’ of the Benchmark should not be used in food preparation, including palm and coconut oil, butter, cream, sour cream, chocolate nut spread and regular coconut milk or cream [14]. Sugary drinks refer to those of no nutritional value with sugar content added during processing, and must not be sold. Dairy beverages are exempted from the sugary drinks [14].

### 2.3. Samples

Thirty-three food outlets were identified through an online search of the food outlets on campus and were visited for the audit. One of them was found to be undergoing renovation, and two refused to participate. Vending machines were assessed with information supplied by a previous audit [18] and visual inspection across the entire campus. A total of 30 food outlets and 62 vending machines were included in this study. 

### 2.4. Data Collection

The food outlet audit was conducted by a visual inspection across the campus to record all available food and drink items in display units (including chilled cabinets, hot food display sections, ambient shelves, fridges and freezers) and menus in each food outlet. Alcoholic beverages sold in a minority of outlets were not recorded in this study. The full names of packaged and freshly prepared foods and drinks were recorded. For packaged products, the HSRs and portion sizes were collected. If the HSR was not available on the package, the nutritional panel and the ingredients list were recorded for HSR calculation. For freshly prepared foods and drinks, the recipes from five outlets were obtained from the major food service provider, including ingredients lists and exact portion sizes. In the remaining outlets, the ingredients lists were recorded if they were displayed on labels and menus. The portion sizes were either estimated by referring to the ‘Visual portion guide’ in the Benchmark [14] or taken from the recipes provided. Samples of cakes, sweet pastries and meat pies were physically weighed to increase the accuracy of estimations for similar products.

To assess the contents in vending machines, an audit tool from Roy et al. [5] was used. For each slot of the vending machines, the name and portion size of the packaged snack or beverage were recorded on a standardised form. The HSRs were also recorded if visible.

### 2.5. Data Analysis

The HSR was calculated for the packaged foods and drinks without HSR via the calculator available from the government website [19]. The calculation was based on the nutritional information from the packages, Australian Food, Supplement and Nutrient Database (AUSNUT) [20] and online resources. The latter two sources were used when information was incomplete from the packages.

For each food outlet and vending machine, the percentages of Everyday and Occasional foods and drinks, choices in ‘does not meet criteria’ categories and sugary drinks were calculated and compared against the Benchmark. To be fully compliant with the Benchmark, a food outlet or a vending machine should contain 75% or more Everyday foods and drinks, less than 25% Occasional choices but no items in ‘does not meet criteria’ categories and no sugary drinks for sale. 

## 3. Results

### 3.1. Food Outlets

A total of 2976 food products and 1309 beverage items were assessed in the food outlet audit. Six types of food outlets were investigated, including stores in food courts (*n* = 7), sandwich outlet (*n* = 1), cafés (*n* = 11), bars and drink stores (*n* = 3), coffee huts (*n* = 5), and convenience stores (*n* = 3).

#### 3.1.1. Packaged Foods

Amongst 536 types of packaged foods in the University food outlets, the proportions of Everyday and Occasional options were 12% and 34%, respectively (Table 2). The remaining choices (54%) failed to meet the criteria for Everyday or Occasional either due to low HSR or large portion sizes.

Of Everyday categories, all dried fruits, canned tuna, and yoghurt met the criteria. Less than half of muesli bars and nuts met the recommendations for both HSR and portion size. Muesli bars with confectionery and nuts with high sodium (i.e., sodium >400 mg) were classified as ‘confectionery’ and ‘salty snacks’ within the Occasional category, respectively. 

Of Occasional categories, a large proportion of sweet biscuits, confectionery, and ice cream exceeded the recommended portion limits. Ninety per cent of salty snacks could not meet the criteria of HSR ≥3.5 due to their high energy and sodium content.

A total of fifteen food outlets offered packaged foods, and they all had less than 20% Everyday options (see Table S1), with an overall proportion of only 8% across campus (see Table 3). The majority of the packaged foods were snacks and stocked in convenience stores.

#### 3.1.2. Freshly Prepared Foods

The overall nutritional quality of freshly prepared foods performed much better than packaged foods as half of the available items were classified as Everyday. Cold meals were the food category with the best quality since 21 out of 23 outlets (91%) achieved the target of 75% Everyday choices (see Table S2), and those under the Occasional category (17%) were mostly sandwiches, salads and sushi with processed or crumbed meat fillings.

The performance of hot meals varied in different outlets as five stores offered 75% or more Everyday hot meals, while this percentage was no more than 25% in another six outlets. Overall, 57% of available hot meals on campus were Everyday. The majority of Occasional foods were offered in large portion sizes that made them unacceptable under the Benchmark, including meat pies, savoury pastries and hot chips.

Cakes, desserts and sweet pastries accounted for most unpackaged snacks and were especially for sale in cafés and coffee huts. Most of them (72%) did not meet the criteria for Occasional foods since their portion sizes exceeded the recommendations. Everyday snacks (14%) in this category were fresh fruits and freshly made yoghurts.

#### 3.1.3. Packaged Beverages

More than half (55%) of available packaged drinks were sugary drinks across all outlets (see Table 3). Permitted Everyday and Occasional beverages only comprised 25% and 10% of available choices, respectively. Moreover, none of the outlets offered more than 44% Everyday packaged drinks (Table S3).

#### 3.1.4. Freshly Made Drinks

Freshly made drinks appeared to be the healthiest selection in food outlets since the proportions of Everyday drinks in all stores were greater than 75% (Table S3). Coffee and tea were mostly Everyday unless too much syrup or flavoured shot was added according to the acquired recipes. Some freshly made drinks were classified as Occasional drinks due to added ice cream in smoothies and milkshakes, and in some cases, this exceeded the permissible portion for Occasional drinks (see Table 3). Two outlets provided sugary drinks including slushies and iced tea with added sugars. 

#### 3.1.5. Overall Compliance with The Benchmark

In general, none of the food outlets were compliant with the Benchmark across both food and drink categories (see Table S4). After combining all food categories, four outlets (14%) that offered Asian, Indian, and Turkish cuisines in food courts appeared to achieve the recommended proportion (75%) for Everyday foods. They rarely used unhealthy ingredients (e.g., processed meat) in cold and hot meals, and offered less Occasional food products such as desserts, meat pies, and hot chips. The overall proportions of Everyday and Occasional foods were 35% versus 22%, and more than 40% of the offerings were not able to meet the criteria which were mostly snack foods (see Table 3).

**Figure 1:**
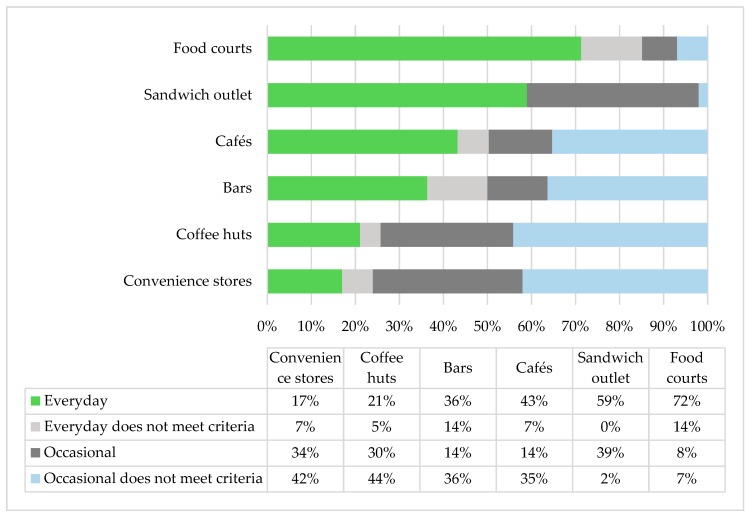
Comparison of food availability by different types of food outlets.

Food classification by different types of outlets is shown in Figure 1. Stores in food courts were the closest to the desired proportion of Everyday foods (72%), including five outlets with ethnic cuisines and two outlets that mainly offered sandwiches and salads. In cafés, bars, and coffee huts, Occasional and ‘does not meet criteria’ choices occupied more than 50% of supplies. Convenience stores appeared to be the least healthy outlet type due to the offering of a wide variety of packaged snacks with poor nutritional quality.

Drinks in two outlets were fully compliant with the Benchmark, and another four stores achieved 75% Everyday drinks but contained sugary drinks. The overall percentage of Everyday drinks was 53%, which was higher than food selections. Nevertheless, this better performance was mainly contributed by freshly made drinks that were mostly Everyday options. Sugary drinks still occupied a third of available choices after combining freshly made drinks with packaged beverages.

### 3.2. Vending Machines

A total of 264 products and 2159 occupied slots were evaluated in the vending machine audit. Three types of vending machines were detected, including machines that stocked snacks only (*n* = 21), beverages only (*n* = 31), and both snacks and drinks (*n* = 10). Two beverage machines were completely compliant with the Benchmark.

Of snack slots, only 9% (*n* = 80) were classified as Everyday, such as muesli bars, crackers with cheese or tuna, and ready-to-eat meals with small portions which met the criteria. Permitted Occasional choices occupied 30% (*n* = 281) of snack slots, and more than 60% were not meeting the criteria for Everyday (6%, *n* = 52) or Occasional (55%, *n* = 511) snacks. The most frequently available Occasional snacks were salty snacks (47%), followed by confectionery (35%). 

Of 1235 beverage slots, 45% were Everyday options that met the criteria, and the most prevalent Everyday drinks were water (27%). Permitted Occasional drinks only occupied 4% of beverage slots as most diet drinks were served in containers larger than 500mL. Sugary drinks occupied 38% of beverage slots across the university. 

Table 4 indicated the snack and beverage classification by different machine vendors. The percentage of Everyday snacks in snack machines that were provided by primary vending contractors was only 2%. Beverage machines contained 43% Everyday options, but 41% sugary drinks. The alternate vendor provided a ‘healthy’ type of machine with 38% of snack slots and 80% of beverage slots as Everyday choices.

## 4. Discussion

Current available food and drink choices in one large university’s food outlets and vending machines were poorly compliant with the Food and Drink Benchmark. The overall nutritional quality of freshly prepared options appeared to be healthier than packaged products, and drinks were better than foods. Across the food environment on campus, better performance was contributed by cold meals and freshly prepared drinks, while packaged snacks, sugary drinks, cakes, meat pies, pastries, and hot chips made it less healthy. Given the high prevalence of overweight and obesity in young adults and their concentration in universities, a specific food and nutrition policy might be helpful to improve the food environment.

Governments across Australia have formed policies to achieve healthier food environments in schools and health facilities with the latter as an exemplar for workplaces [13,14,21]. However, universities have no mandatory restriction regarding the quality and promotion of available foods and drinks. Health promotion activities aimed at individual behaviour change must be accompanied by reshaping of the food environment. Policy should be developed for such restructuring to support the healthy choice to be the easy choice. While the proportional change required to reduce excessive energy intake by young adults is uncertain, increasing the availability of healthy foods is one of the top priorities recommended by the McKinsey Global Institute report as a strategy to combat obesity [22].

Potential opportunities for interventions based on our study findings could be delivered to policy-makers for universities and other tertiary education institutions. First, sugary drinks occupied more than half of the available packaged choices in cafeterias and more than a third in vending machines. The removal of sugary drinks was the first step to implement the Framework in health facilities [14]. The causal relationship between sugary drinks consumption and obesity, and its association with type 2 diabetes and other chronic diseases have been indicated from a meta-analysis [23]. More than 90% of university students and staff were aware of the health risk of sugary drinks according to an online survey but did not support complete removal [24]. Reduction in the current proportion of sugary drinks and possible price incentives for healthier drinks might nudge them towards healthier choices. It is possible that when healthier drinks predominate and with continual exposure that they will become the new norm [25]. 

Second, there is a wide gap between the current proportion of Everyday foods and the recommendation from existing policy which was developed with the specific intention to be implemented in health facilities. Similar benchmarks could be established for universities but may need to be adapted to produce the desired change in improving the quality and energy density of student diets. The Benchmarks in the university setting might not need to be so stringent around the proportions of Everyday and Occasional food choices given young adults may have higher energy requirements than middle-aged and older adults. It seems a feasible approach would be to produce a stepwise improvement in the proportions, e.g., start from at least 50% Everyday choices in each category in consultation with the food service providers. Aiming to increase the varieties and facings of Everyday foods might be easier than persuading retailers to remove ‘does not meet criteria’ choices that may contribute the most to the revenue of the stores. The reduction of portion sizes and restriction on ingredients could be implemented in a later stage when contractors become more willing to be involved. According to the experience from the implementation in hospitals, strong language such as ‘ban’ and ‘prohibit’ may stimulate negative responses from food retailers [14]. 

Although the Benchmark classifies 99% fruit juice without added sugar as an Everyday drink [14], juices contain higher energy and less fibre when compared with whole fruits [15]. Therefore, to avoid excessive juice consumption on an individual basis, future guidelines for food outlets and vending machines in universities may need to require provision for more access to fresh fruits and water and less promotion of juices. 

A significant proportion of food outlets in tertiary education institutions are supplied by external contractors, which may require more effort on negotiation to address the balance between public health and commercial profits [26]. Food outlets in new buildings could be a unique opportunity to apply the policy since it allows interventions in the stage of choosing suppliers and recipe modifications to make healthy choices readily available from the beginning. Moreover, partnerships with the food service vendors and other experienced organisations may facilitate the implementation.

The majority of current vending machines were stocked by several large snack and beverage companies and offered a limited proportion of healthy products. The nutritional quality of snacks and beverages from the machines provided by an alternate vendor was closer to the recommendations. There are also some other healthier vending brands in the market across Australia [27,28], and signing contracts with these companies is recommended when current contracts near expiration [14]. 

Apart from changing food offerings, the following strategies may be included to restructure the university food environment. Food marketing and promotions should feature Everyday foods and be restricted for Occasional and other foods and sugary drinks [14]. Young adults have often been targeted by marketing campaigns but less prioritised by public health policies [29], and exposure to food advertising may negatively influence their diets [30]. Lower prices for healthier food choices were often suggested by the public, and may have positive impacts on purchasing behaviours [8,24,31]. Another strategy might be placing Everyday foods and drinks at prominent locations, such as checkout areas, and eye level of shelving units and vending machines [14]. The effectiveness of nutritional labels on menus was inconsistent from previous studies. However, additional labels on pre-packaged products in a university setting had a small but significant impact on healthier food choices [32,33].

This study follows from a previous audit of seven tertiary institutions in NSW [5]. While a different benchmarking system was applied, the predominance of unhealthy packaged snacks for sale and sugary beverages is similar to the findings of the current study. Follow-up monitoring at regular intervals should be implemented in concert with policy changes. 

The current study has several strengths and limitation in its methods. The nutritional analysis of recipes, HSR calculation, and in person visual inspection of outlets including their menus, foods, and beverages for sale gives legitimacy to the findings. To make better estimations and assumptions, a qualified dietitian weighed food samples and checked recipes from either the AUSNUT database [20] or traditional recipes online, when the portion sizes and/or complete recipes were not available for freshly prepared items. The audits focused on product availability, quality, and size, and, thus, the current circumstances of food marketing including the cost, promotion, and positioning were not assessed. These strategies would be incorporated in the policy design and implementation as well as follow-up studies.

## 5. Conclusions

The overall compliance of current university food outlets and vending machines with the existing Food and Drink Benchmark is poor. Removing sugar-sweetened beverages should be the initial focus of making changes for the university food environment, followed by increasing the facings of Everyday choices and reducing the portion sizes of Occasional foods and drinks to meet the criteria. Detailed communication with stakeholders (e.g., food service sectors and contractors) regarding problems and solutions and more efforts in marketing will be necessary to implement the changes. To improve the overall quality and promotion of foods and drinks in the universities, government policy should be developed to mandate the actions that required for this specific setting.

## Figures and Tables

**Table 1:** Foods and drinks classification criteria.

Classification	Food Categories	Meets Criteria	Does Not Meet Criteria
**Snacks**			
**Everyday**	**Muesli and snack bars**	No confectionery, HSR ≥ 3.5 stars, portion size ≤ 50 g)	No confectionery, HSR < 3.5 stars and/or portion size > 50 g
	**Nuts, seeds, legume snacks and popcorn**	a. unsalted b. lightly salted (sodium ≤ 400 mg), no sweet coatings or confectionery, HSR ≥ 3.5 stars, portion size ≤ 50 g	Lightly salted (sodium ≤ 400 mg), no sweet coatings or confectionery, HSR < 3.5 stars and/or portion size > 50 g
	**Savoury biscuits/crackers**	Crackers (HSR ≥ 3.5 stars) with dips, cheese and tuna	Crackers (HSR < 3.5 stars) with cheese
	**Dried fruit**	Portion size ≤ 50 g	Portion size > 50 g
	**Fruit**	Fresh fruit	NA ^1^
	**Yoghurt**	No added confectionery	NA
	**Canned tuna**	a. Canned tuna b. Tuna/Salmon with pasta	NA
**Occasional**	**Salty snacks** e.g., chips and crisps, flavoured baked savoury biscuits, salted nuts and seeds (sodium > 400 mg)	HSR ≥ 3.5 stars, portion size ≤ 50 g	HSR < 3.5 stars and/or portion size > 50 g
	**Sweet biscuits**	Portion size ≤ 50 g	Portion size > 50 g
	**Confectionery** e.g., Chocolate, lollies, chewing gum, products containing confectionery (e.g., muesli bars)	Portion size ≤ 50 g	Portion size > 50 g
	**Cakes and sweet pastries** e.g., muffins, banana bread, slices, croissants	Portion size ≤ 80 g	Portion size > 80 g
	**Desserts** e.g., mousse, cheesecake	Portion size ≤ 100 g	Portion size > 100 g
	**Ice cream, frozen yoghurt**	Portion size ≤ 85 mL	Portion size > 85 mL
**Cold meals**			
**Everyday**	**Sandwiches/wraps/rolls, sushi/ rice paper rolls, salads, frittata**	Without Occasional foods and fillings (see below)	Without Occasional foods and fillings, but use cream/sour cream
**Occasional**	**Sandwiches/wraps/rolls, sushi/ rice paper rolls, salads, frittata**	With Occasional foods and fillings: processed meat ≤ 60 g, coated/crumbed meat ≤ 140 g	With Occasional foods and fillings: processed meat > 60 g, coated/crumbed meat > 140 g, and/or use cream/sour cream
**Hot meals**			
**Everyday**	**Toasties and open melts, soup, pasta, pizza, risotto and rice dishes, Indian, Asian, Mexican, jacket potatoes, burger, dim sum, hot breakfast**	Without Occasional foods and fillings (see below)	Without Occasional foods and fillings, but use butter/cream /sour cream/chocolate nut spread/coconut milk
**Occasional**	**Toasties and open melts, soup, pasta, pizza, risotto and rice dishes, Indian, Asian, Mexican, jacket potatoes, burger, dim sum, hot breakfast**	With Occasional foods and fillings (within portion limits), and portion size of entire meal ≤ 450 g	With Occasional foods and fillings: fillings exceed portion limits, and/or portion size of entire meal > 450 g, and/or use butter/cream /sour cream/ chocolate nut spread/coconut milk
	**Pies**	Portion size ≤ 180 g (potato pie ≤ 250 g)	Portion size > 180 g (potato pie > 250 g)
	**Sausage rolls/savoury pastries**	Portion size ≤ 120 g	Portion size > 120 g
	**Instant flavoured noodles**	HSR ≥ 3.5 stars, portion size ≤ 75 g (dry pack weight)	HSR < 3.5 stars and/or portion size > 75 g (dry pack weight)
**Occasional foods and fillings**	**Processed meat (served hot or cold)** e.g., bacon, salami, pepperoni, chorizo, prosciutto, sausages	Portion size ≤ 60 g	Portion size > 60 g
	**Coated/crumbed meat (served hot or cold)** e.g., chicken nuggets, schnitzels, fish fingers, kibbeh	Portion size ≤ 140 g	Portion size > 140 g
	**Hot potato products** e.g., hot chips, wedges	Portion size ≤ 100 g	Portion size > 100 g
	**Corn chips or hard taco shells**	Portion size ≤ 50 g	Portion size > 50 g
	**Garlic bread, cheese and bacon rolls**	Portion size ≤ 90 g	Portion size 90 g
**Drinks**			
**Everyday**	**Water, milk and milk alternatives, tea**	No added sugars or intense sweeteners	
	**Coffee**	Use no more than 1 level tablespoon of flavouring powder or 20 mL syrup per portion, no added cream, portion size ≤ 500 mL	With added cream, and/or use more than 1 level tablespoon of flavouring powder or 20 mL syrup per portion, and/or portion size > 500 mL
	**99% Fruit/vegetable juice/coconut water**	No added sugars or intense sweeteners, portion size ≤ 400 mL	No added sugars or intense sweeteners, portion size > 400 mL
	**Flavoured milk and milk alternatives, liquid breakfast drinks/protein drinks**	Portion size 500 mL, HSR ≥ 3.5 stars (if packaged), the same criteria as ‘coffee’ if freshly prepared	Portion size > 500 mL, HSR < 3.5 stars (if packaged), the same criteria as ‘coffee’ if freshly prepared
	**Milkshakes/smoothies** (with no added ice cream/gelato/sorbet)	Portion size ≤ 500 mL	Portion size > 500 mL
**Occasional**	**Thick shakes/smoothies** (with added ice cream/gelato/sorbet)	Portion size ≤ 500 mL, added ice cream/gelato/sorbet ≤ 125 mL	Portion size > 500 mL, and/or added ice cream/gelato/sorbet > 125 mL
	**Diet and sugar-free drinks** (drinks with added intense sweeteners, no added sugar)	Portion size ≤ 500 mL	Portion size > 500 mL
**Sugary drinks**	Drinks with added sugars and no nutritional value (this excludes milk drinks)	e.g., soft drinks, energy drinks, flavoured water, sports drinks, fruit drinks, slushies, coconut water with added sugar	

^1^ NA: not applicable.

**Table 2:** Available packaged food product types in food outlets on campus (*n* = 536) ^1^.

Classification	Food Categories	Meets Criteria % (*n*)	Does Not Meet Criteria % (*n*)	Total % (*n*)
Everyday	Muesli and snack bars	41 (29)	59 (41)	13 (70)
	Nuts and seeds, legume snacks	45 (10)	55 (12)	4 (22)
	Savoury biscuits/crackers	80 (4)	20 (1)	1 (5)
	Dried fruit/ yoghurt/tuna/cereal	100 (19)	0 (0)	4 (19)
	**Total**	**12 (62)**	**10 (54)**	**22 (116)**
Occasional	Salty snacks	10 (10)	90 (87)	18 (97)
	Instant noodles	0 (0)	100 (11)	2 (11)
	Sweet biscuits/sweet pastry	25 (5)	75 (15)	4 (20)
	Confectionery	61 (154)	39 (98)	47 (252)
	Ice cream	35 (14)	65 (26)	7 (40)
	**Total**	**34 (183)**	**44 (237)**	**78 (420)**

^1^ Percentages in total rows and columns are percentages within total types, while other percentages are within specific food categories.

**Table 3:** Foods and drinks classification across all 30 audited food outlets.

Categories	Items % (*N*)				
	Everyday	Everyday Does Not Meet Criteria	Occasional	Occasional Does Not Meet Criteria	Sugary Drinks (Drinks Only)
**Foods (*n* = 2976)**					
Packaged foods	8 (88)	9 (96)	39 (412)	44 (463)	NA ^1^
Freshly prepared foods overall	50 (968)	6 (122)	13 (244)	30 (583)	NA
Cold meals	82 (402)	0 (1)	17 (83)	1 (4)	NA
Hot meals	57 (489)	14 (121)	9 (80)	20 (175)	NA
Unpackaged snacks	14 (77)	0 (0)	14 (81)	72 (404)	NA
**Total**	**35 (1056)**	**7 (218)**	**22 (656)**	**35 (1046)**	**NA**
**Drinks (*n* = 1309)**					
Packaged drinks	25 (185)	6 (42)	10 (76)	5 (38)	55 (409)
Freshly prepared drinks	92 (513)	1 (6)	3 (18)	1 (4)	3 (18)
**Total**	**53 (698)**	**4 (48)**	**7 (94)**	**3 (42)**	**33 (427)**

^1^ NA: not applicable.

**Table 4:** Foods and drinks classification across all 30 audited food outlets.

Vending Machines	Everyday	Everyday Does Not Meet Criteria	Occasional	Occasional Does Not Meet Criteria	Sugary Drinks (Drinks Only)
**Snacks**					
Primary Snack Machines	2%	3%	34%	61%	NA ^1^
‘Healthy’ machines	38%	17%	13%	33%	NA
Others ^2^	4%	6%	33%	57%	NA
**Beverages**					
Beverage Machines	43%	2%	2%	12%	41%
‘Healthy’ machines	80%	4%	15%	0%	0%
Others ^2^	32%	2%	16%	2%	48%

^1^ NA: not applicable. ^2^ Machines (other than the ‘healthy’ machines) that contained both foods and drinks.

## Supplementary Materials

The following are available online at http://www.mdpi.com/2072-6643/10/12/1909/s1, Table S1: The classification of available packaged and freshly prepared foods in food outlets across the campus, Table S2: The classification of available freshly prepared foods (i.e., cold meals, hot meals, and unpackaged snacks) in food outlets across the campus, Table S3: The classification of available packaged and freshly prepared drinks in food outlets across the campus, Table S4: The classification of available foods and drinks in food outlets across the campus.
