# Supplementary material for: It Is Time to Make Policy for Healthier Food Environments in Australian Universities
Source: Nutrients. 2018 Dec 4;10(12):1909. doi: 10.3390/nu10121909 (PMC6316519; doi:10.3390/nu10121909)
Supplement: Supplementary file 1 [file nutrients-10-01909-s001.pdf]

Table S1. The classification of available packaged and freshly prepared foods in food outlets across the campus

Food outlet types	No.	Packaged foods, n (%)				Freshly prepared foods, n (%)			
		Everyday	Everyday does not meet criteria	Occasional	Occasional does not meet criteria	Everyday	Everyday does not meet criteria	Occasional	Occasional does not meet criteria
Food courts	1	NA [1]	NA	NA	NA	30 (79)	0 (0)	3 (8)	5 (13)
	2	NA	NA	NA	NA	21 (75)	1 (4)	6 (21)	0 (0)
	3	NA	NA	NA	NA	43 (84)	6 (12)	2 (4)	0 (0)
	4	NA	NA	NA	NA	45 (87)	6 (12)	1 (2)	0 (0)
	5	NA	NA	NA	NA	21 (44)	20 (42)	4 (8)	3 (6)
	6	NA	NA	NA	NA	13 (72)	5 (28)	0 (0)	0 (0)
	7	NA	NA	NA	NA	28 (62)	0 (0)	6 (13)	11 (24)
Sandwich outlet	8	NA	NA	NA	NA	35 (59)	0 (0)	23 (39)	1 (2)
Cafés	9	0 (0)	2 (33)	2 (33)	2 (33)	28 (27)	2 (2)	19 (19)	53 (52)
	10	0 (0)	1 (33)	1 (33)	1 (33)	57 (43)	8 (6)	13 (10)	56 (42)
	11	0 (0)	2 (67)	1 (33)	0 (0)	50 (31)	23 (14)	9 (6)	79 (49)
	12	NA	NA	NA	NA	8 (29)	6 (21)	4 (14)	10 (36)
	13	0 (0)	0 (0)	4 (40)	6 (60)	43 (44)	4 (4)	15 (15)	35 (36)
	14	1 (4)	0 (0)	14 (50)	13 (46)	69 (63)	6 (5)	14 (13)	21 (19)
	15	1 (6)	2 (12)	9 (53)	5 (29)	87 (58)	21 (14)	13 (9)	29 (19)
	16	NA	NA	NA	NA	37 (39)	2 (2)	4 (4)	51 (54)
	17	NA	NA	NA	NA	54 (52)	4 (4)	21 (20)	25 (24)
	18	NA	NA	NA	NA	37 (62)	0 (0)	6 (10)	17 (28)
	19	3 (6)	5 (10)	20 (42)	20 (42)	105 (57)	5 (3)	23 (13)	50 (27)
Bars/drinks	20	NA	NA	NA	NA	4 (31)	1 (8)	2 (15)	6 (46)
	21	NA	NA	NA	NA	4 (44)	2 (22)	1 (11)	2 (22)
	22	NA	NA	NA	NA	NA	NA	NA	NA
Coffee huts	23	6 (7)	8 (9)	46 (53)	27 (31)	10 (36)	0 (0)	4 (14)	14 (50)
	24	3 (5)	8 (14)	14 (24)	33 (57)	25 (41)	0 (0)	8 (13)	28 (46)
	25	1 (5)	3 (14)	8 (38)	9 (43)	23 (40)	0 (0)	12 (21)	23 (40)
	26	2 (6)	1 (3)	17 (49)	15 (43)	18 (35)	0 (0)	12 (24)	21 (41)
	27	4 (17)	1 (4)	5 (21)	14 (58)	0 (0)	0 (0)	1 (14)	6 (86)
Convenience stores	28	6 (4)	6 (4)	71 (49)	63 (43)	NA	NA	NA	NA
	29	38 (13)	26 (9)	101 (34)	132 (44)	43 (66)	0 (0)	8 (12)	14 (22)
	30	23 (8)	31 (11)	99 (36)	123 (45)	30 (48)	0 (0)	10 (16)	23 (37)

[1] NA: not available

Table S2. The classification of available freshly prepared foods (i.e. cold meals, hot meals and unpackaged snacks) in food outlets across the campus

Food outlet types	No.	Cold meals, n (%)				Hot meals, n (%)				Unpackaged snacks, n (%)			
		Everyday	Everyday does not meet criteria	Occasional	Occasional does not meet criteria	Everyday	Everyday does not meet criteria	Occasional	Occasional does not meet criteria	Everyday	Everyday does not meet criteria	Occasional	Occasional does not meet criteria
Food courts	1	6 (100)	0 (0)	0 (0)	0 (0)	24 (75)	0 (0)	3 (9)	5 (16)	NA [1]	NA	NA	NA
	2	3 (100)	0 (0)	0 (0)	0 (0)	18 (78)	1 (4)	4 (17)	0 (0)	0 (0)	0 (0)	2 (100)	0 (0)
	3	4 (100)	0 (0)	0 (0)	0 (0)	39 (83)	6 (13)	2 (4)	0 (0)	NA	NA	NA	NA
	4	23 (100)	0 (0)	0 (0)	0 (0)	22 (76)	6 (21)	1 (3)	0 (0)	NA	NA	NA	NA
	5	3 (100)	0 (0)	0 (0)	0 (0)	18 (42)	20 (47)	4 (9)	1 (2)	0 (0)	0 (0)	0 (0)	2 (100)
	6	5 (100)	0 (0)	0 (0)	0 (0)	5 (50)	5 (50)	0 (0)	0 (0)	3 (100)	0 (0)	0 (0)	0 (0)
	7	19 (83)	0 (0)	4 (17)	0 (0)	3 (38)	0 (0)	2 (25)	3 (38)	6 (43)	0 (0)	0 (0)	8 (57)
Sandwich outlet	8	33 (67)	0 (0)	16 (33)	0 (0)	1 (25)	0 (0)	3 (75)	0 (0)	1 (17)	0 (0)	4 (67)	1 (17)
Cafés	9	17 (74)	0 (0)	5 (22)	1 (4)	4 (21)	2 (11)	3 (16)	10 (53)	7 (12)	0 (0)	11 (18)	42 (70)
	10	23 (92)	0 (0)	0 (0)	2 (8)	27 (51)	8 (15)	2 (4)	16 (30)	7 (13)	0 (0)	11 (20)	38 (68)
	11	8 (89)	0 (0)	0 (0)	1 (11)	33 (53)	23 (37)	0 (0)	6 (10)	9 (10)	0 (0)	9 (10)	72 (80)
	12	NA	NA	NA	NA	8 (38)	6 (29)	4 (19)	3 (14)	0 (0)	0 (0)	0 (0)	7 (100)
	13	19 (83)	0 (0)	4 (17)	0 (0)	19 (42)	4 (9)	6 (13)	16 (36)	5 (17)	0 (0)	5 (17)	19 (66)
	14	27 (84)	0 (0)	5 (16)	0 (0)	37 (65)	6 (11)	3 (5)	11 (19)	5 (24)	0 (0)	6 (29)	10 (48)
	15	15 (79)	0 (0)	4 (21)	0 (0)	69 (67)	21 (20)	7 (7)	6 (6)	3 (11)	0 (0)	2 (7)	23 (82)
	16	4 (67)	1 (17)	1 (17)	0 (0)	28 (50)	1 (2)	3 (5)	24 (43)	5 (16)	0 (0)	0 (0)	27 (84)
	17	NA	NA	NA	NA	51 (62)	4 (5)	17 (21)	10 (12)	3 (14)	0 (0)	4 (18)	15 (68)
	18	8 (100)	0 (0)	0 (0)	0 (0)	23 (77)	0 (0)	2 (7)	5 (17)	6 (27)	0 (0)	4 (18)	12 (55)
	19	56 (79)	0 (0)	15 (21)	0 (0)	45 (59)	5 (7)	6 (8)	20 (26)	4 (11)	0 (0)	2 (6)	30 (83)
Bars/drinks	20	NA	NA	NA	NA	4 (31)	1 (8)	2 (15)	6 (46)	NA	NA	NA	NA
	21	NA	NA	NA	NA	3 (60)	2 (40)	0 (0)	0 (0)	1 (25)	0 (0)	1 (25)	2 (50)
	22	NA	NA	NA	NA	NA	NA	NA	NA	NA	NA	NA	NA
Coffee huts	23	10 (77)	0 (0)	3 (23)	0 (0)	0 (0)	0 (0)	0 (0)	6 (100)	0 (0)	0 (0)	1 (11)	8 (89)
	24	20 (80)	0 (0)	5 (20)	0 (0)	0 (0)	0 (0)	0 (0)	9 (100)	5 (19)	0 (0)	3 (11)	19 (70)
	25	17 (85)	0 (0)	3 (15)	0 (0)	4 (36)	0 (0)	3 (27)	4 (36)	2 (7)	0 (0)	6 (22)	19 (70)
	26	14 (82)	0 (0)	3 (18)	0 (0)	4 (36)	0 (0)	3 (27)	4 (36)	0 (0)	0 (0)	6 (26)	17 (74)
	27	NA	NA	NA	NA	NA	NA	NA	NA	0 (0)	0 (0)	1 (14)	6 (86)
Convenience stores	28	NA	NA	NA	NA	NA	NA	NA	NA	NA	NA	NA	NA
	29	38 (83)	0 (0)	8 (17)	0 (0)	0 (0)	0 (0)	0 (0)	2 (100)	5 (29)	0 (0)	0 (0)	12 (71)
	30	30 (81)	0 (0)	7 (19)	0 (0)	0 (0)	0 (0)	0 (0)	8 (100)	0 (0)	0 (0)	3 (17)	15 (83)

[1] NA: not available

Table S3. The classification of available packaged and freshly prepared drinks in food outlets across the campus

Food outlet types	No.	Packaged drinks, n (%)					Freshly prepared drinks, n (%)				
		Everyday	Everyday does not meet criteria	Occasional	Occasional does not meet criteria	Sugary drinks	Everyday	Everyday does not meet criteria	Occasional	Occasional does not meet criteria	Sugary drinks
Food courts	1	4 (12)	0 (0)	4 (12)	2 (6)	24 (71)	NA [1]	NA	NA	NA	NA
	2	7 (14)	1 (2)	3 (6)	3 (6)	35 (71)	3 (100)	0 (0)	0 (0)	0 (0)	0 (0)
	3	3 (12)	2 (8)	1 (4)	2 (8)	18 (69)	NA	NA	NA	NA	NA
	4	4 (16)	1 (4)	2 (8)	2 (8)	16 (64)	NA	NA	NA	NA	NA
	5	NA	NA	NA	NA	NA	15 (88)	0 (0)	2 (12)	0 (0)	0 (0)
	6	NA	NA	NA	NA	NA	12 (80)	0 (0)	3 (20)	0 (0)	0 (0)
	7	7 (29)	0 (0)	3 (13)	2 (8)	12 (50)	NA	NA	NA	NA	NA
Sandwich outlet	8	3 (17)	4 (22)	0 (0)	2 (11)	9 (50)	5 (100)	0 (0)	0 (0)	0 (0)	0 (0)
Cafés	9	6 (43)	0 (0)	6 (43)	0 (0)	2 (14)	26 (100)	0 (0)	0 (0)	0 (0)	0 (0)
	10	5 (31)	0 (0)	7 (44)	0 (0)	4 (25)	30 (100)	0 (0)	0 (0)	0 (0)	0 (0)
	11	3 (23)	0 (0)	4 (31)	1 (8)	5 (38)	28 (76)	4 (11)	2 (5)	3 (8)	0 (0)
	12	2 (17)	1 (8)	1 (8)	0 (0)	8 (67)	30 (97)	0 (0)	1 (3)	0 (0)	0 (0)
	13	3 (15)	0 (0)	2 (10)	0 (0)	15 (75)	27 (96)	0 (0)	1 (4)	0 (0)	0 (0)
	14	7 (21)	0 (0)	2 (6)	1 (3)	24 (71)	29 (97)	0 (0)	0 (0)	0 (0)	1 (3)
	15	5 (11)	6 (14)	3 (7)	1 (2)	29 (66)	36 (86)	2 (5)	4 (10)	0 (0)	0 (0)
	16	7 (16)	2 (5)	3 (7)	1 (2)	31 (70)	29 (100)	0 (0)	0 (0)	0 (0)	0 (0)
	17	0 (0)	0 (0)	0 (0)	2 (33)	4 (67)	26 (93)	0 (0)	1 (4)	1 (4)	0 (0)
	18	2 (33)	0 (0)	0 (0)	2 (33)	2 (33)	8 (89)	0 (0)	1 (11)	0 (0)	0 (0)
	19	15 (25)	8 (13)	3 (5)	2 (3)	32 (53)	9 (75)	0 (0)	3 (25)	0 (0)	0 (0)
Bars/drinks	20	NA	NA	NA	NA	NA	NA	NA	NA	NA	NA
	21	NA	NA	NA	NA	NA	NA	NA	NA	NA	NA
	22	NA	NA	NA	NA	NA	56 (77)	0 (0)	0 (0)	0 (0)	17 (23)
Coffee huts	23	7 (27)	3 (12)	0 (0)	2 (8)	14 (54)	21 (100)	0 (0)	0 (0)	0 (0)	0 (0)
	24	17 (43)	0 (0)	4 (10)	2 (5)	17 (43)	30 (100)	0 (0)	0 (0)	0 (0)	0 (0)
	25	8 (26)	2 (6)	6 (19)	2 (6)	13 (42)	19 (100)	0 (0)	0 (0)	0 (0)	0 (0)
	26	12 (36)	2 (6)	0 (0)	2 (6)	17 (52)	19 (100)	0 (0)	0 (0)	0 (0)	0 (0)
	27	8 (44)	0 (0)	0 (0)	1 (6)	9 (50)	20 (100)	0 (0)	0 (0)	0 (0)	0 (0)
Convenience stores	28	7 (19)	0 (0)	7 (19)	2 (5)	21 (57)	NA	NA	NA	NA	NA
	29	15 (33)	0 (0)	7 (16)	2 (4)	21 (47)	16 (100)	0 (0)	0 (0)	0 (0)	0 (0)
	30	28 (37)	10 (13)	8 (11)	2 (3)	27 (36)	19 (100)	0 (0)	0 (0)	0 (0)	0 (0)

[1] NA: not available

Table S4. The classification of available foods and drinks in food outlets across the campus

Food outlet types	No.	Total foods, n (%)				Total drinks, n (%)				
		Everyday	Everyday does not meet criteria	Occasional	Occasional does not meet criteria	Everyday	Everyday does not meet criteria	Occasional	Occasional does not meet criteria	Sugary drinks
Food courts	1	30 (79)	0 (0)	3 (8)	5 (13)	4 (12)	0 (0)	4 (12)	2 (6)	24 (71)
	2	21 (75)	1 (4)	6 (21)	0 (0)	10 (19)	1 (2)	3 (6)	3 (6)	35 (67)
	3	43 (84)	6 (12)	2 (4)	0 (0)	3 (12)	2 (8)	1 (4)	2 (8)	18 (69)
	4	45 (87)	6 (12)	1 (2)	0 (0)	4 (16)	1 (4)	2 (8)	2 (8)	16 (64)
	5	21 (44)	20 (42)	4 (8)	3 (6)	15 (88)	0 (0)	2 (12)	0 (0)	0 (0)
	6	13 (72)	5 (28)	0 (0)	0 (0)	12 (80)	0 (0)	3 (20)	0 (0)	0 (0)
	7	28 (62)	0 (0)	6 (13)	11 (24)	7 (29)	0 (0)	3 (13)	2 (8)	12 (50)
Sandwich outlet	8	35 (59)	0 (0)	23 (39)	1 (2)	8 (35)	4 (17)	0 (0)	2 (9)	9 (39)
Cafés	9	28 (26)	4 (4)	21 (19)	55 (51)	32 (80)	0 (0)	6 (15)	0 (0)	2 (5)
	10	57 (42)	9 (7)	14 (10)	57 (42)	35 (76)	0 (0)	7 (15)	0 (0)	4 (9)
	11	50 (30)	25 (15)	10 (6)	79 (48)	31 (62)	4 (8)	6 (12)	4 (8)	5 (10)
	12	8 (29)	6 (21)	4 (14)	10 (36)	32 (74)	1 (2)	2 (5)	0 (0)	8 (19)
	13	43 (40)	4 (4)	19 (18)	41 (38)	30 (63)	0 (0)	3 (6)	0 (0)	15 (31)
	14	70 (51)	6 (4)	28 (20)	34 (25)	36 (56)	0 (0)	2 (3)	1 (2)	25 (39)
	15	88 (53)	23 (14)	22 (13)	34 (20)	41 (48)	8 (9)	7 (8)	1 (1)	29 (34)
	16	37 (39)	2 (2)	4 (4)	51 (54)	36 (49)	2 (3)	3 (4)	1 (1)	31 (42)
	17	54 (52)	4 (4)	21 (20)	25 (24)	26 (76)	0 (0)	1 (3)	3 (9)	4 (12)
	18	37 (62)	0 (0)	6 (10)	17 (28)	10 (67)	0 (0)	1 (7)	2 (13)	2 (13)
	19	108 (47)	10 (4)	43 (19)	70 (30)	24 (33)	8 (11)	6 (8)	2 (3)	32 (44)
Bars/drinks	20	4 (31)	1 (8)	2 (15)	6 (46)	NA [1]	NA	NA	NA	NA
	21	4 (44)	2 (22)	1 (11)	2 (22)	NA	NA	NA	NA	NA
	22	NA	NA	NA	NA	56 (77)	0 (0)	0 (0)	0 (0)	17 (23)
Coffee huts	23	16 (14)	8 (7)	50 (43)	41 (36)	28 (60)	3 (6)	0 (0)	2 (4)	14 (30)
	24	28 (24)	8 (7)	22 (18)	61 (51)	47 (67)	0 (0)	4 (6)	2 (3)	17 (24)
	25	24 (30)	3 (4)	20 (25)	32 (41)	27 (54)	2 (4)	6 (12)	2 (4)	13 (26)
	26	20 (23)	1 (1)	29 (34)	36 (42)	31 (60)	2 (4)	0 (0)	2 (4)	17 (33)
	27	4 (13)	1 (3)	6 (19)	20 (65)	28 (74)	0 (0)	0 (0)	1 (3)	9 (24)
Convenience stores	28	6 (4)	6 (4)	71 (49)	63 (43)	7 (19)	0 (0)	7 (19)	2 (5)	21 (57)
	29	81 (22)	26 (7)	109 (30)	146 (40)	31 (51)	0 (0)	7 (11)	2 (3)	21 (34)
	30	53 (16)	31 (9)	109 (32)	146 (43)	47 (50)	10 (11)	8 (9)	2 (2)	27 (29)

[1] NA: not available
